# Adult Colocolic Intussusception: A Rare Case of Intestinal Obstruction

**DOI:** 10.7759/cureus.39526

**Published:** 2023-05-26

**Authors:** Joana Frazão, Carolina Silva, Filipe Almeida, José Calado, Paulo Mira

**Affiliations:** 1 General Surgery, Hospital Prof. Doutor Fernando Fonseca, Lisboa, PRT; 2 General Surgery, Centro Hospitalar do Oeste, Caldas da Rainha, PRT

**Keywords:** oncological ressection, adult intussusception, colon cancer, colocolic intussusception, intestinal obstruction

## Abstract

Intussusception occurs when a part of the intestine slides into its distal adjacent portion and is a surgical emergency. Adult colocolic intussusception is rare, but it is a severe condition and is usually associated with a presence of a tumoral process. We present the case of a frail male patient admitted to our emergency department with abdominal pain, prostration, and dyspnea. The patient was diagnosed with colocolic intussusception and was submitted to a subtotal colectomy and ileostomy. Patients with colocolic intussusception usually present with chronic abdominal pain and signs of intestinal obstruction. Abdominal CT scan facilitates the diagnosis, but most cases are only diagnosed intraoperatively. Given the high probability of colon cancer, the treatment involves an oncological resection of the intestinal segment. Colocolic intussusception is a rare cause of intestinal obstruction in adults where a high suspicion index is of paramount importance, especially considering that most of the diagnoses are made at surgery.

## Introduction

Intussusception occurs when a proximal segment of the gastrointestinal tract telescopes into the lumen of an adjacent segment, causing an intestinal obstruction [1-5]. Although quite common in children in the first two years of life, it is rare among adults, accounting for only 5% of all intussusceptions. In adults, intussusceptions represent only 1%-5% of all cases of intestinal obstruction and are usually associated with a definable cause, with 50%-75% of colocolic intussusceptions having a tumor as its lead point [1-3,5-8]. Until obstruction occurs, diagnosis is difficult due to its unspecific symptoms and requires a high index of suspicion [1,2,5]. Abdominal CT is the modality of choice for diagnosing intussusception. As the leading cause of intussusceptions in adults is colon cancer, surgical resection is the preferred treatment choice in this age group [2-6].

In this case report, we discuss a case of colocolic intussusception caused by a descendent colon adenocarcinoma.

## Case presentation

An 81-year-old frail male patient, partially dependent and with a known history of dementia associated with Parkinson's disease, was presented to our emergency department with abdominal pain, constipation, prostration, and dyspnea.

On examination, the patient was afebrile, emaciated, and reactive to stimuli but confused. His blood pressure and pulse rate were normal. He needed 2L/min of supplementary oxygen to be eupneic. His abdomen was tympanized, distended, and painful, particularly in the right flank, without rebound tenderness or guarding.

**Figure 1:**
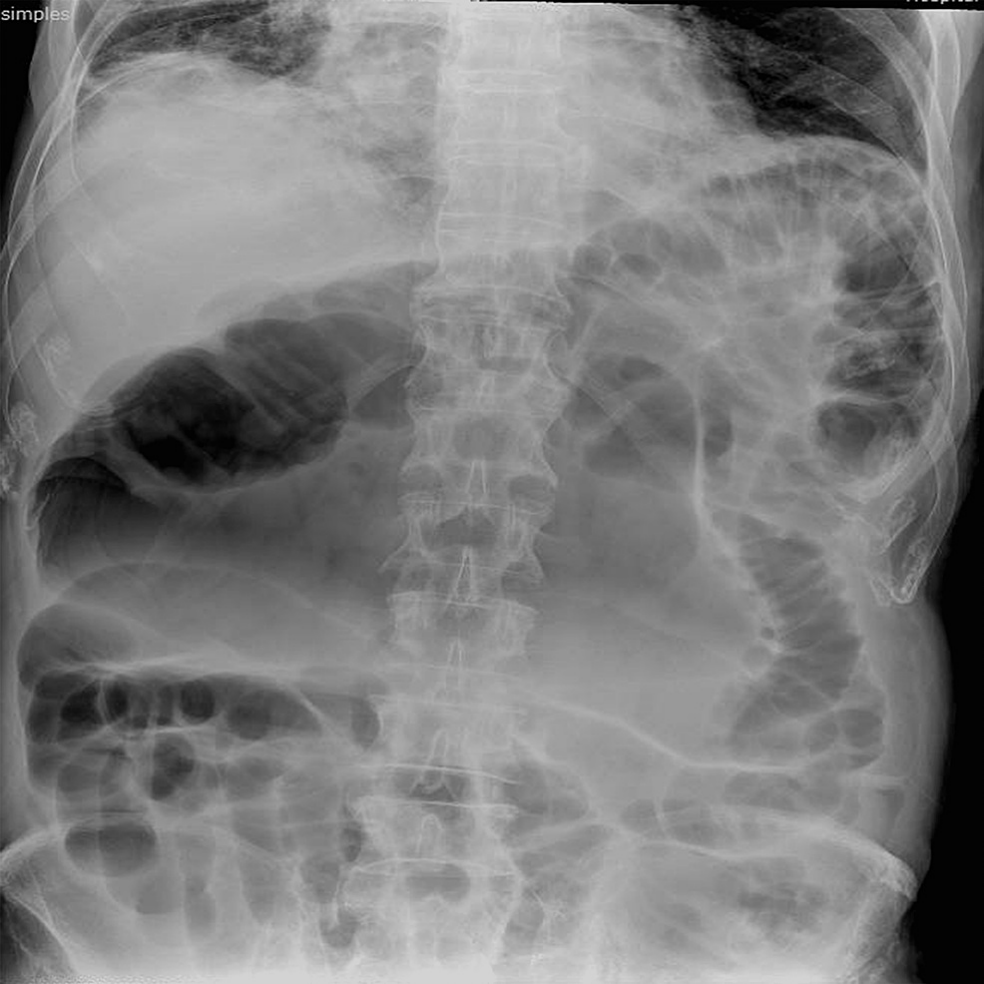
Abdominal X-ray revealing dilated large bowel loops

**Figure 2:**
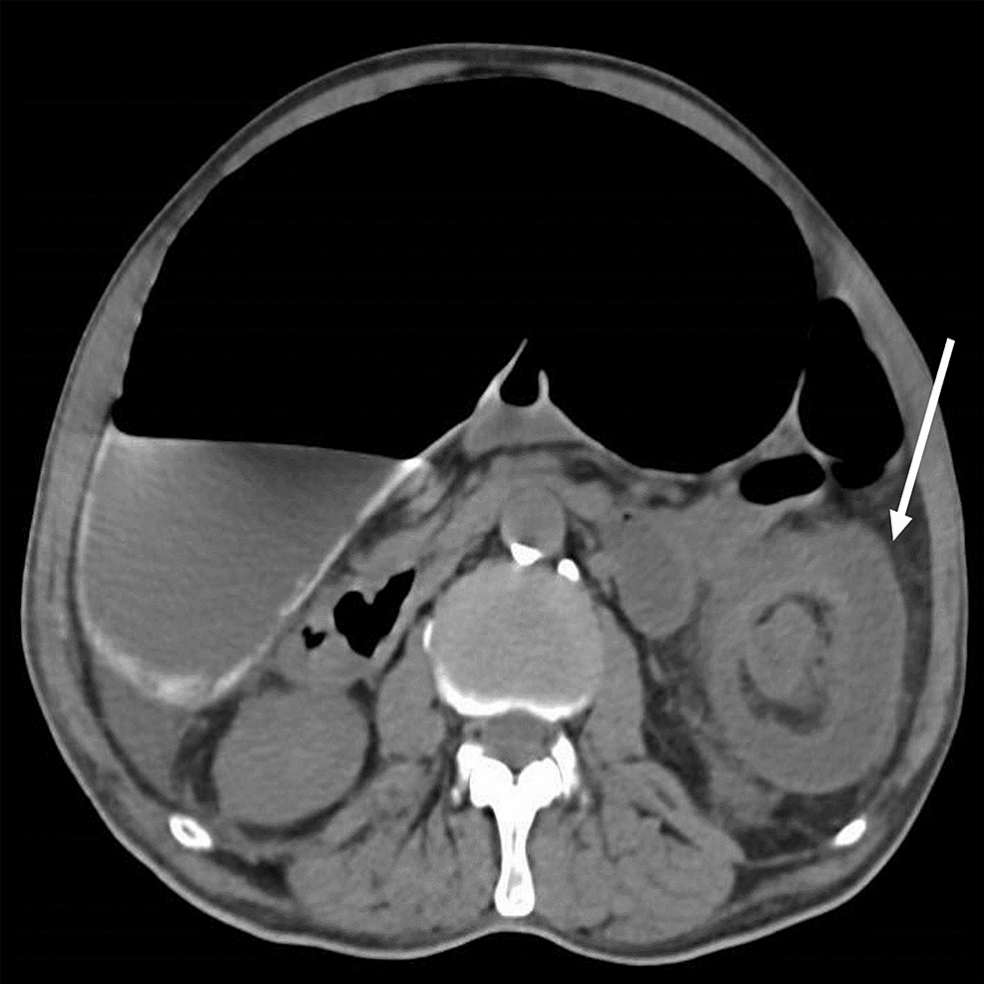
Abdominal CT scan showing a classic target sign, suggestive of a colocolic intussusception

Laboratory tests were within the normal range. A thoracic X-ray revealed a right base condensation compatible with pneumonia. The abdominal X-ray showed dilated large bowel loops (Figure 1). An abdominal CT scan showed bilateral pulmonary consolidations and a left colocolic intussusception with the classic target sign, minimal free fluid, and no complication signs (Figure 2). These findings established the diagnosis of an intestinal obstruction caused by colocolic intussusception.

**Figure 3:**
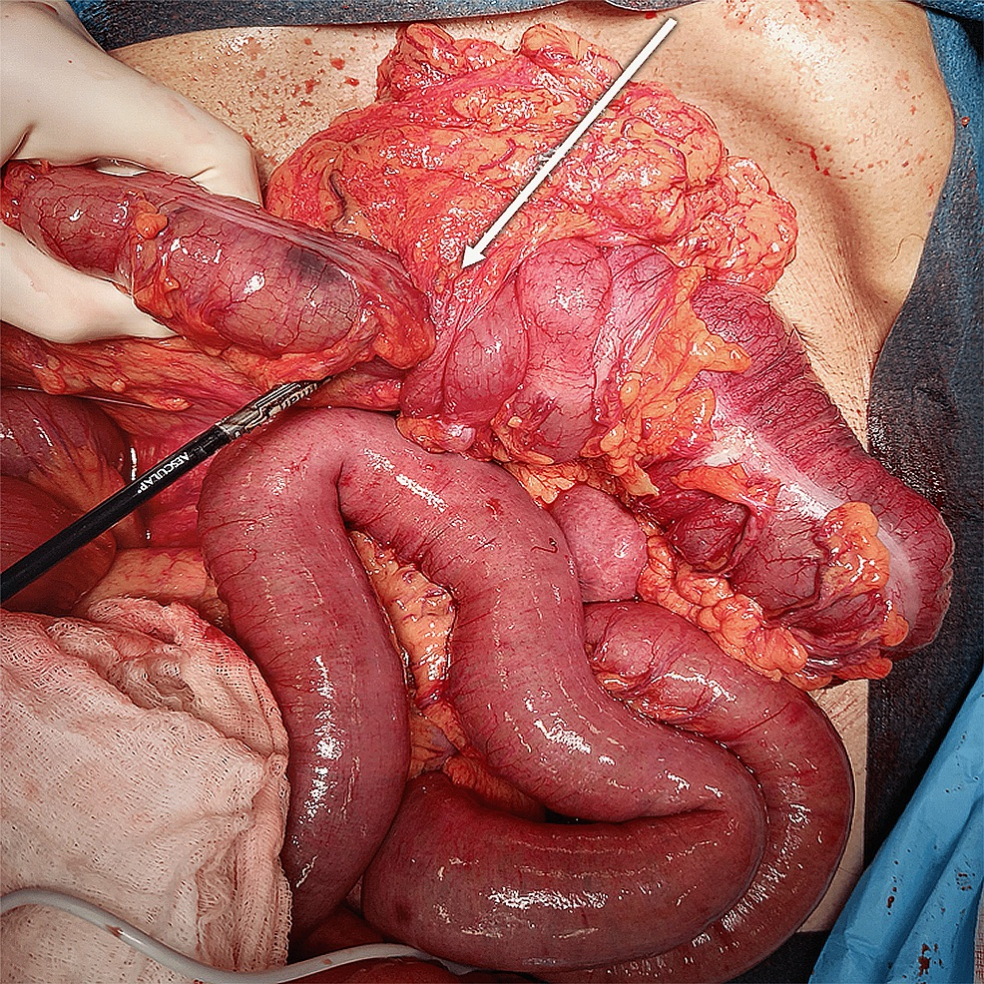
Intra-operative image: large bowel loop intussusception extending into the descending colon

**Figure 4:**
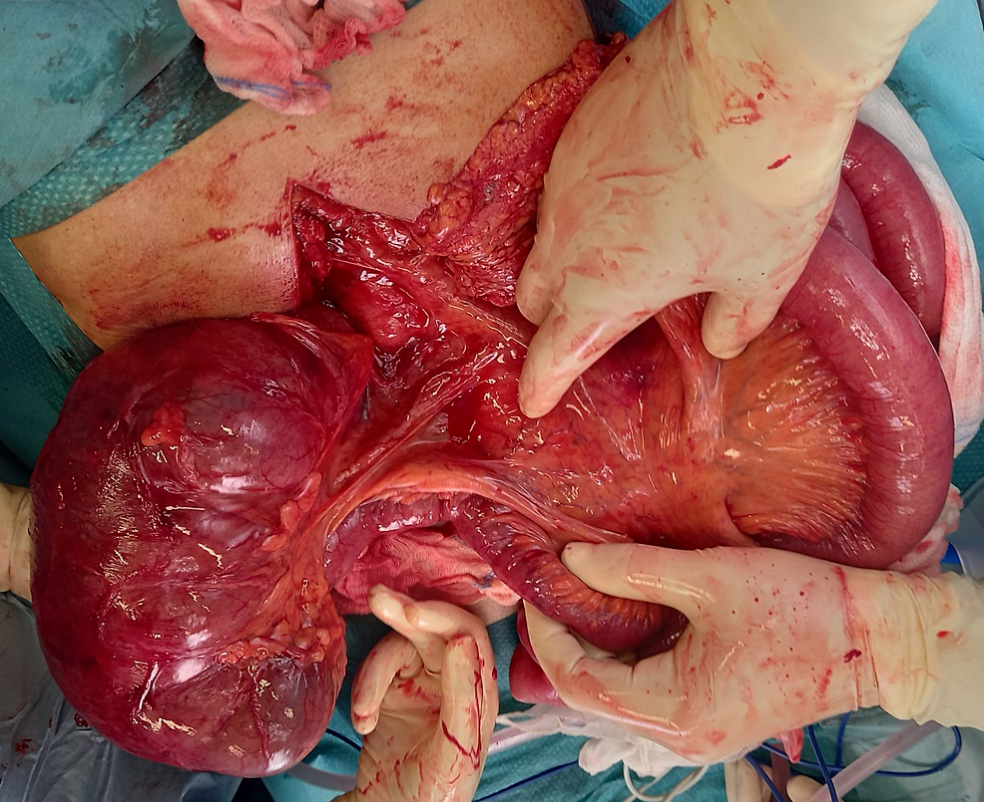
Intra-operative image: caecum diastasis

**Figure 5:**
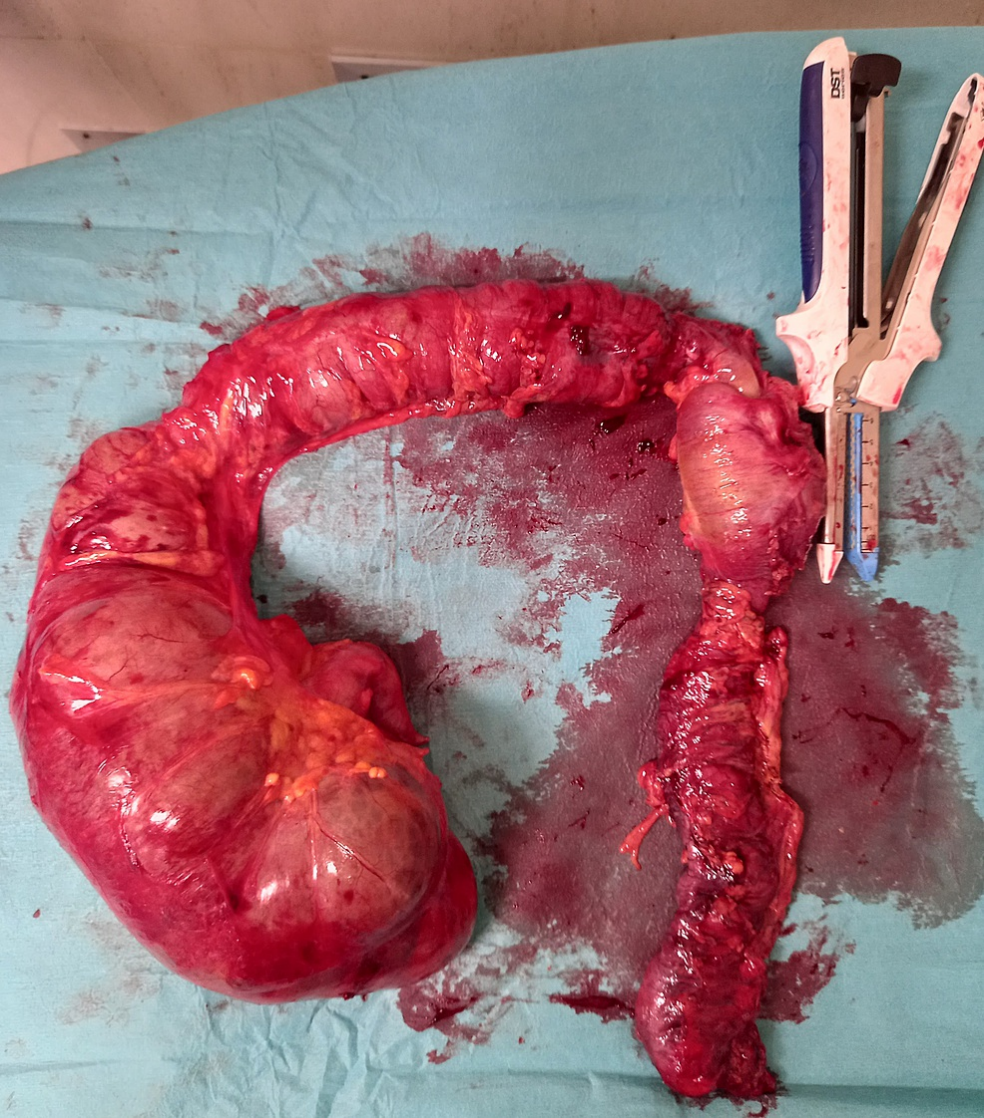
Specimen of subtotal colectomy

The patient was promptly given antibiotics and intravenous fluids and underwent nasogastric decompression and exploratory laparotomy. At laparotomy, a large bowel loop intussusception was found (of over 10 cm in length) extending into the descending colon with a colonic mass as the lead point of the invagination (Figure 3). The proximal bowel loops were distended conditioning a caecum diastasis (Figure 4). As the patient was fragile, a subtotal colectomy with terminal ileostomy and a mucous fistula was performed (Figure 5). 

Histopathological findings revealed an adenocarcinoma on a tubulovillous adenoma with a high and low grade of dysplasia, 0/14 lymph nodes, pT2N0R0.

Because of the pneumonia, the patient needed to be ventilated at the ICU for two days, with progressive improvement. After that, he recovered well and was discharged. On follow-up, the patient is doing well and is free of the disease.

## Discussion

Intussusception is a rare cause of intestinal obstruction in adults, representing 1%-5% of bowel obstructions and 0.003%-0.02% of all hospital admissions [5,7]. This condition can be classified according to its location, etiology (idiopathic, benign, or malignant), and the presence or absence of a lead point. Among the three types of gastrointestinal intussusceptions based on location, colocolic intussusceptions (like the case presented here) are the least prevalent in adults, whereas ileocaecal are the most common, followed by enteroenteric intussusceptions [3,5,7].

Unlike children, idiopathic cases account for only 8% to 10% of adult intussusceptions. Most (90%) adult intussusceptions have a definable cause, 50% to 75% of which are malignancies [2,3,5,8].

The diagnosis of intussusception is complex and rarely made preoperatively since its clinical symptoms are atypical and unspecific. Most patients usually present with obstructive symptoms, chronic abdominal pain, and a palpable mass [2,5,6,8]. Its complications, such as bowel obstruction, bleeding, infarction, perforation, or peritonitis, may change the clinical presentation [8]. Radiological investigations are extremely helpful for diagnosis. Abdominal X-rays, though not sensitive or specific, can show multiple air-fluid levels and a site of intestinal obstruction [1,2,3,5]. Ultrasonography can be helpful by visualizing the classical "target sign". However, the gas fluid-filled intestinal loops, and obesity are its major limiting factors [2,3,5,8]. Abdominal CT scan plays a central role in the intussusception diagnosis because it is the most sensitive study for this condition. The characteristic features of CT scan include a nonhomogeneous "target" shaped soft tissue mass with a layering effect and mesenteric vessels within the bowels' lumen. CT scan can also localize the intussusception and evaluate the presence and degree of complications [2,5-8].

Early surgical intervention is the treatment modality of choice in adults due to the high occurrence of bowel ischemia and gangrene [5,6,8]. Since most cases are caused by underlying colon cancer, an oncological resection should be performed. Due to the risk of perforation and dissemination of tumoral cells, reduction of the intussusception is not advised [2,3,5-8]. The decision between primary anastomosis or stoma confection depends on the location of the intussusception, bowel wall integrity, degree of contamination, hemodynamic status of the patient, and the surgeon's preference [2,8]. In this case, a hugely dilated caecum with impending perforation was uncovered during laparotomy. Therefore, we decided against performing an anastomosis. Moreover, the fact that the patient was old and extremely fragile supported this decision.

## Conclusions

Adult colocolic intussusception is a rare condition that affects adults and, in most cases, is secondary to colon carcinoma. Nonspecific symptoms make preoperative diagnosis difficult, and a high index of suspicion is needed. Most patients are diagnosed only during the exploratory laparotomy. Preoperative radiology, especially the CT scan, facilitates diagnosis. Operative intervention is required, and an en-bloc resection is the best approach, given the high risk of malignancy.
